# Yoga as a modality in occupational therapy practice for adults experiencing mood disorders: A systematic review and meta-analysis

**DOI:** 10.1177/03080226251412253

**Published:** 2026-01-30

**Authors:** Tenzin Dolker, Tenzin Dolma, Adrianna Spoerel, Marley Cameron, Briana Bortolin, Anna Colebatch, Candice Crooks, Helena Toolsiedas, Javier Mencia Ledo, Bao-Zhu Stephanie Long, Sana Siddiqui, Raihana Premji, Alicia McDougall, Behdin Nowrouzi-Kia

**Affiliations:** 1 Department of Occupational Science and Occupational Therapy, University of Toronto, Toronto, ON, Canada; 2 Restore Lab, Department of Occupational Science and Occupational Therapy, Temerty Faculty of Medicine, University of Toronto, Toronto, ON, Canada; 3 Centre for Research in Occupational Safety and Health, Laurentian University, Sudbury, ON, Canada; 4 Institute for Mental Health Policy Research, Centre for Addiction and Mental Health, Toronto, ON, Canada

**Keywords:** Mood disorders, yoga, major depressive disorder, bipolar disorder, occupational therapy

## Abstract

**Introduction:**

Yoga is a non-pharmacological intervention that promotes holistic health and helps manage clinical symptoms. There has been growing interest in integrating yoga into occupational therapy to address specific disorders. This systematic review aims to evaluate the current literature on yoga and mood disorders, identifying the potential use of yoga in occupational therapy to manage symptoms and support occupational participation in adults experiencing mood disorders.

**Methods:**

Six databases were searched. Data were extracted from 22 peer-reviewed articles published between 2002 and 2024 using the Population, Intervention, Comparison and Outcomes framework. The Critical Appraisal Skills Programme checklist was used for quality appraisal of included studies. The ‘metafor package’ was used to conduct a meta-analysis and generate a forest plot for data synthesis. An *I*^2^ statistic was used to quantify heterogeneity of the meta-analysis, and the trim and fill non-parametric method was used for sensitivity analyses.

**Results:**

Yoga has a moderate positive effect in supporting symptom management (i.e. depressive and anxiety symptoms) and daily functioning, including cognitive functioning, of adults experiencing mood disorders.

**Conclusion:**

Yoga can mitigate barriers to occupational participation for adults with mood disorders through supporting symptom management. Rigorous studies are needed to establish yoga’s effects on mood disorders.

## Introduction

### Overview of yoga and mood disorders

Yoga, a mindful and physical practice originating from ancient Indian philosophy, emphasizes promoting holistic health and well-being (Garfinkel and Schumacher, 2000; National Center for Complementary and Integrative Health, 2023). The physical exercises in yoga postures help to increase strength, balance and coordination, while the mindful techniques help enhance awareness and concentration (Chen, 2024). By incorporating physical postures, breathing exercises and meditation techniques, yoga aims to enhance an individual’s physical, mental and spiritual well-being (Thomas et al., 2021). Yoga therapy utilizes the foundational principles of yoga to promote mind–body wellness within a therapeutic context for clients (Pearson et al., 2020). The International Association of Yoga Therapists (2024) has defined the therapeutic use of yoga as the ‘process of empowering individuals to progress toward improved health and well-being through the application of the teachings and practices of yoga’.

The American Occupational Therapy Association has recognized yoga as a complementary integrative therapy with multiple benefits, including improvements in strength, flexibility and balance, as well as an enhancement in quality of life (Park and Slattery, 2021; Rose et al., 2021). Furthermore, the National Center for Complementary and Integrative Health (2023) has also recognized yoga as a form of complementary alternative and integrative medicine, a non-traditional intervention used to manage clinical symptoms (Ng et al., 2023).

In 2019, it was estimated that 280 million people and 40 million people live with depression and bipolar disorder, respectively worldwide (World Health Organization, 2000). In Canada alone, it was estimated that 3 million Canadians over the age of 18 were living with a mood and/or anxiety disorder in 2013, with over one-quarter of these adults reporting that this has affected their lives and limited their engagement in daily activities (Government of Canada, 2015). Additionally, 50% of these individuals required work accommodations, and 35% terminated their work due to their symptoms (Government of Canada, 2015). Furthermore, in 2022, it was reported that the prevalence of mood and anxiety disorders in Canadians over the age of 15 has increased substantially over the last 10 years (Statistic Canada, 2023).

Mood disorders can be generally described as a category of mental health conditions that share the common feature of ‘prolonged, pervasive emotional disturbance’ (American Psychological Association, 2023). According to the fifth edition of the *Diagnostic Statistical Manual* (DSM-5), mood disorders can be classified as either depressive or bipolar disorders, each of which has its subtypes with unique diagnostic criteria and characteristics (*Diagnostic and Statistical Manual of Mental Disorders: DSM-5*, 2013). The subtypes of depressive disorders include major depressive disorder, persistent depressive disorder and premenstrual dysphoric disorder; whereas bipolar disorder subtypes include bipolar I disorder, bipolar II disorder and cyclothymic disorder (*Diagnostic and Statistical Manual of Mental Disorders: DSM-5*, 2013). Since some individuals with mood disorders such as major depressive disorders experience treatment resistance to traditional therapeutic options, it is important to explore alternative treatments to support better symptom management and functioning (Voineskos et al., 2020).

### Mood disorders and occupations

Occupations are described as meaningful activities or tasks that a person engages in during their daily life, occupying their time (Townsend and Polatajko, 2013). These are activities that people want to do, need to do or are expected to do. Occupations include self-care (e.g. brushing teeth, eating), productive (e.g. work, volunteering) and leisure activities (i.e. activities that individuals engage in for enjoyment; Angner et al., 2013; Canadian Association of Occupational Therapists, 2023; Law, 2002). Symptoms of mood disorders impact everyday functioning, decrease motivation to engage in activities and hinder social interactions (Gould et al., 2015; Jean et al., 2022; Kupferberg and Hasler, 2023). As such, depressive disorders have been reported to increase difficulty in fulfilling social and occupational roles in all three domains of self-care, productivity and leisure (Gunnarsson et al., 2023). Therefore, occupational therapists, who are holistic healthcare providers, can collaborate on treatment interventions with adults experiencing mood disorders to address barriers to their occupational participation.

### The use of yoga in occupational therapy practice

Occupational therapy is a holistic, client-centred field that focuses on enabling people of all ages to engage in or re-engage with daily activities, promoting health and well-being (Hammond, 2004; Law, 2002; World Federation of Occupational Therapists, 2024). This uniquely positions occupational therapists to serve their clients’ mental, physical, spiritual and environmental needs, thereby improving their occupational engagement (Canadian Association of Occupational Therapists, 2025; Johnston and Mayers, 2005; Kirsh et al., 2019). Similarly, a fundamental principle of yoga is a holistic approach to health, aimed at alleviating pain on several similar domains (Madan et al., 2023). Yoga is particularly suitable in occupational therapy practice when it is understood as a meaningful occupation, involving purposeful engagement to improve health and well-being (Townsend and Polatajko, 2013). Additionally, it is commonly undertaken in group settings, serving as a co-occupation with significant health implications through social connection (Smith and Atencio, 2017).

Recently, more research has explored the use of yoga in occupational therapy practice with different populations. For example, recent findings have provided supporting evidence for the effectiveness of implementing yoga within a therapeutic context, with notable preliminary effects in decreasing the severity of depressive, anxiety and other mood disorder symptoms (Moosburner et al., 2024; Park and Slattery, 2021).

Similarly, yoga interventions have had significant positive effects in patients with a history of stroke (Schmid et al., 2016), Parkinsons’s disease (Hill et al., 2020), chronic brain injury (Stephens et al., 2020) and diabetic peripheral neuropathy (Willis Boslego et al., 2017). Several studies have found improvements in balance, fall risk, self-efficacy, activity participation and fatigue management, supporting overall symptom management in populations with existing health challenges (Schmid et al., 2016). Furthermore, yoga interventions have been shown to reduce occupational barriers for individuals with chronic pain, brain injury and neuropathy, improving the quality and quantity of occupational engagement and increasing job satisfaction (Rose et al., 2021; Schmid et al., 2019; Stephens et al., 2020; Willis Boslego et al., 2017).

Further research has shown that occupational therapists tend to have positive views on the integration of yoga therapy due to its client-centred nature and ability to be tailored to meet the unique needs of individual clients, ultimately promoting a holistic approach to health and well-being that is effective across various populations (Andrews et al., 2021; Graham and Plummer, 2018).

Although results from the current literature suggest that yoga has the potential to be a beneficial modality in occupational therapy practice for different populations, more research is still needed to explore this with individuals experiencing mood disorders. In addition, the current literature on yoga has reported concerns regarding poor methodology and rigour, particularly regarding the use of small sample sizes (Bridges and Sharma, 2017; Jean et al., 2022), thus, resulting in a scarcity of high-quality studies (Gupta and Dhawan, 2022). Overall, research examining the effectiveness of yoga in symptom management for mood disorders has yielded promising findings; however, more rigorous studies are needed to understand its therapeutic effects better (Kumar et al., 2019; Wu et al., 2023).

### Purpose of this systematic review

Despite the growing literature supporting the integration of yoga into therapeutic contexts, more research is needed on the effectiveness of incorporating yoga into occupational therapy practice for adults with mood disorders. This systematic review aims to evaluate the current literature on yoga and mood disorders, identifying the potential use of yoga in occupational therapy practice to manage symptoms and support occupational participation in adults experiencing mood disorders.

## Methods

### Overview

This systematic review follows the Preferred Reporting Items for Systematic Reviews and Meta-Analyses (PRISMA) framework (Page et al., 2021). A systematic review protocol for this review is publicly accessible via PROSPERO registration number CRD42021283157, which outlines the review objectives, eligibility criteria and planned methods (Crooks et al., 2024).

### Eligibility criteria

The eligibility criteria for this study were established using the Population, Intervention, Comparisons, Outcomes (PICO) framework (Richardson et al., 1995). The inclusion and exclusion criteria were developed to ensure that the studies included were relevant and would directly address our research question. Quantitative data from peer-reviewed journal articles were selected for the study according to the following criteria.

#### Inclusion criteria

Included studies were peer-reviewed and published in or translated into English. The studies included must have participants diagnosed with a mood disorder, as defined in the *Diagnostic and Statistical Manual of Mental Disorders-4* (DSM-4) or DSM-5, or the *International Classification of Diseases-10* (ICD-10) or ICD-11. Only studies using adult participants over the age of 18 were included. Only articles published within 22 years of the search were included (2002–2024) to ensure clinical significance and applicability.

#### Exclusion criteria

Studies were excluded if the participants were not diagnosed with a mood disorder according to the DSM-4 or DSM-5 or ICD-10 or ICD-11. Studies were also excluded if participants were younger than 18 years old. Additionally, qualitative studies and non-peer-reviewed articles (e.g. grey literature and white papers) were also excluded to maintain methodological consistency and ensure the collection of data that is suitable for meta-analysis. Knowledge syntheses, including scoping, systematic and narrative reviews, were excluded. Studies published outside of the 22-year timeframe from 2002 to 2024 were excluded.

### Information sources and search strategy

The research team searched six online databases, guided by a health systems librarian, to retrieve relevant studies. The healthcare databases used were OVID Medline, Embase, CINAHL Plus, Cochrane Library, APA PsycINFO and Scopus. The search terms ‘yoga’ and ‘yogic’ were used interchangeably for the intervention, as they pertain to related concepts. Search terms regarding mood disorders were generated to include the different types of bipolar and depressive disorders such as bipolar I disorder, bipolar II disorder, major depressive disorder, persistent depressive disorder, chronic depressive disorder, chronic depression, premenstrual dysphoric disorder and cyclothymic disorder (Supplemental Appendix A).

### Selection process

Following the search, articles retrieved from various databases were imported into Covidence (2024), a systematic review management software platform, to remove duplicates. A title and abstract screening were first performed by two independent reviewers (B.B. and A.C.) to ensure all studies were relevant and followed the eligibility criteria. This screening process was first piloted to ensure the two reviewers’ agreement. After this confirmation, they manually screened the remaining studies independently. The same reviewers then subsequently performed a full-text review to examine the potentially relevant studies against the eligibility criteria more thoroughly. The reasons for exclusion were documented in Covidence. All disagreements between reviewers were resolved through discussion.

**Figure 1.**
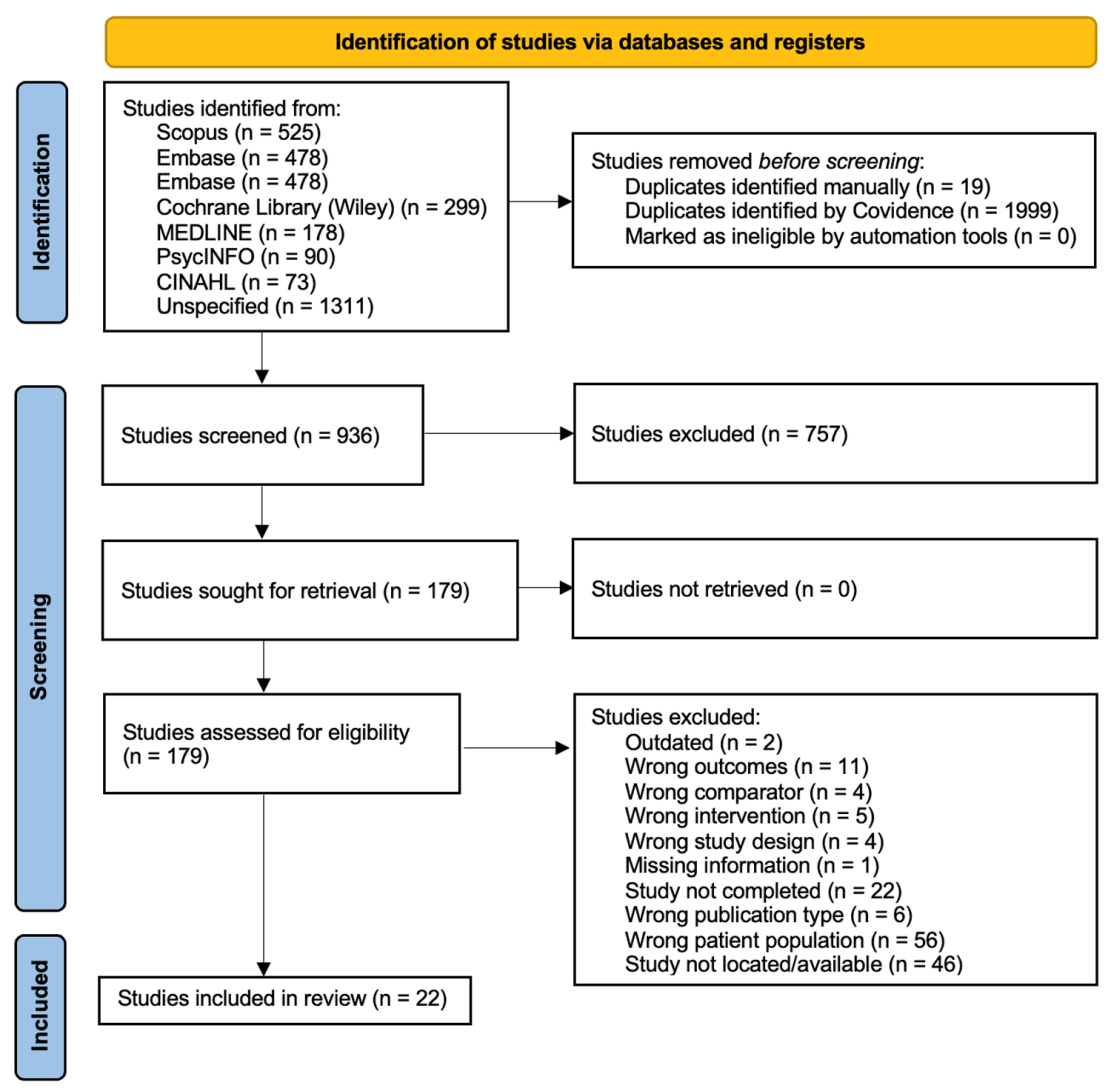
PRISMA flow diagram. PRISMA: Preferred Reporting Items for Systematic Reviews and Meta-Analyses.

Two reviewers (T.Da. and T.Db.) conducted an updated search and screening of studies in October 2024 using the same search strategies previously developed in 2022. Moreover, the reviewers completed the title, abstract and full-text screening to include any relevant articles that may have been published after the initial screening process. All reasons for exclusion were documented in Covidence, and disagreements between the two reviewers were resolved through consultation with the senior author (BNK). Inter-rater reliability was not formally calculated as consensus was achieved for all included studies. The full study selection processes is outlined in the PRISMA flow diagram (Figure 1).

### Data collection process

Data from the studies included in the systematic review were extracted by six independent reviewers from the research team using a data extraction form created based on the PICO framework. Data on the following were collected: study characteristics (study identifiers and study design), sample characteristics (demographic and diagnostic information), intervention characteristics (type of yoga, dosage, frequency, total intervention length, etc.), comparator or control characteristics (control intervention, dosage, frequency, etc.), outcomes (primary and secondary) and results (pre- and post-intervention measure of outcomes and pre- and post-intervention standard deviations of those scores). Data extraction was equally distributed between six reviewers. Each reviewer independently input the information extracted from the articles into a Microsoft Excel sheet to organize the collected data. Any disagreements between reviewers were resolved through consultation with the senior author (BNK).

### Study risk of bias assessment

The Critical Appraisal Skills Programme (CASP) checklist for randomized controlled trials was used to assess the risk of bias for each of the selected articles. The CASP is a critical appraisal checklist with 11 questions used to systematically evaluate the study design, methodology, results, and the applicability of the findings to the local context. Two raters (A.S. and M.C.) independently assessed the risk of bias for all included studies and documenting their findings, including rationales, in Covidence. All disagreements between the two raters were resolved through discussion and consultation with the research team. As a result, consultation with the larger research team was not necessary to reach a consensus. As the CASP checklist does not produce a final score, the authors developed and employed a modified scoring system, consistent with previous methodological reviews (Barr and Tsai, 2021; Bertini et al., 2022). In this system, ‘yes’ responses were interpreted as indicating a low risk of bias and were thus assigned a score of one. Furthermore, ‘can’t tell’ and ‘no’ responses were deemed to have unclear and high risk of bias, respectively; therefore, they both received a score of zero. The three sub-questions regarding blinding under question four of the CASP checklist were graded individually to ensure transparency. As a result, studies were given an overall score of 13, where a score above 8 is considered to be a low risk of bias, a score between 5 and 7 is determined to be an unclear risk of bias, and a score below 4 indicates a high risk of bias.

### Effect measures

The standard mean difference (SMD) was chosen as the effect measure for this review, as the data collected from the studies consisted of quantitative pre- and post-intervention scores, along with their standard deviations. Also, 95% confidence intervals (CIs) were given for each outcome score. Eight of the 30 studies were excluded from the meta-analysis as they did not report complete standard deviation scores. As a result, primary and/or secondary outcomes from 22 studies were included in the data synthesis portion of this review.

### Synthesis methods

Two reviewers (T.Da. and T.Db.) summarized the characteristics and criteria of the included studies. From the synthesized data, a meta-analysis was conducted using the ‘metafor package’ of R Version (4.5.1 for Windows; R Core Team, 2024) to analyse the overall effect of yoga on occupational engagement for adults with mood disorders. A random effects model was employed to account for the expected diversity of factors present within the individual studies. Additionally, a forest plot was generated to combine the results from the synthesized articles where the primary and/or secondary outcomes were combined into a single column. A Higgins I*I*^2^ statistic and τ^2^ models were used to quantify and measure the heterogeneity of the meta-analysis. Additionally, a fail-safe N analysis was conducted to assess the robustness of the synthesized data and determine the number of studies with a null effect that would be required in the meta-analysis to render the overall results statistically non-significant. Lastly, the trim and fill non-parametric method was used to complete sensitivity analyses and account for publication bias.

## Results

### Study selection

A total of 936 articles were identified after removing the duplicates. Following the title and abstract screening, 757 studies were excluded due to failure to meet the inclusion criteria. The remaining 179 articles underwent full-text screening to assess their eligibility, of which 157 were excluded. In total, 22 articles met the final inclusion criteria and were included in this review. Figure 1 presents the PRISMA flow diagram illustrating the full screening process.

### Results of individual studies

A summary of the study characteristics is seen in Table 1. Additionally, Table 2 presents the summary of the results of the included articles.

**Table 1.** Characteristics of included studies.

Author (year), country	Study design; sample size	Mood disorder	Intervention(s)	Intervention duration	Control
Bieber et al. (2021), Germany	RCT, parallel; *n* = 83	MDD	Ashtanga-yoga + TAU	90 minutes, 3×/week, 12-weeks total	Antidepressant medication + psychotherapy
Brinsley et al. (2022), Australia	5-arm randomized controlled crossover trial, crossover; *n* = 41	Depression and anxiety disorders	Yin yoga + Vinyasa yoga + aerobic exercise (stationary cycling) + stretching	30 minutes, 1×/week, 6.5-weeks total (mean time)	No intervention
Butler et al. (2008), USA	Randomized pilot study, parallel; *n* = 46	Diagnosis with a depressive disorder: dysthymia, double (dysthymia and Major Depressive Episode (MDE)), chronic major depression, partial MDE	Hatha yoga + psychoeducation, group therapy + hypnosis + psychoeducation	120 + 30 minutes (home practice), 1×/week, 12-weeks total 90 minutes, 1×/week, 12-week total	Psychoeducation
Field et al. (2013), USA	RCT, parallel; *n* = 92	Dysthymia or MDD	Yoga (unspecified)	20 minutes, 1×/week, 12-week total	Social support group
Halappa et al. (2018), India	Single-blind comparative trial, three groups, parallel; *n* = 84	MDD	Yoga therapy (unspecified), yoga therapy + medication	60 minutes, 7×/week (yoga training) + 60 minutes, 2×/week (yoga therapy) + 2 months (home practice), 12-week total	Medication alone, healthy control
Vollbehr et al. (2022), Netherlands	RCT, parallel; *n* *=* 171	MDD	MYI + mindful instructions	90 minutes, 1×/week + 30–40 minutes (home practice), 9-week total	TAU
La Rocque et al. (2021), Canada	RCT, parallel; *n* *=* 53	Unipolar depressive disorder	Bikram yoga	90 minutes, 2×/week, 8-week total	Aerobic exercises, waitlist control
O’Shea et al. (2023), Australia	Mixed method, parallel; *n* *=* 59	Depressive or anxiety disorder	CBT + therapeutic yoga	120 minutes (CBT) + 60 minute (yoga therapy) + 15–30-minute (home practice), 1×/week, 8-week total	CBT
Ravindran et al. (2021), Canada	RCT, cross-over; *n* = 72	MDD, dysthymia, bipolar disorder	Breathing-focused yoga (unspecified)	90 minutes, 2×/week, 8-week total	Psychoeducation
Schuver and Lewis (2016), USA	Prospective, randomized controlled intervention pilot study, parallel; *n* *=* 40	Depression	Mindfulness-based yoga + mindfulness telephone sessions	60–75 minutes mindfulness-based yoga, 2×/week + 15 minute telephone, 1–3×/week, 12-week total	Walking group + telephone sessions
Scott et al. (2019), USA	RCT, parallel; *n* *=* 30	MDD	Iyengar yoga + coherent breathing (high-dose group)	90-minute yoga, 3×/week + 30-minute (home practice), 4/week, 12-week total	Iyengar yoga + coherent breathing (low-dose group)
Sharma et al. (2006), India	RCT, parallel; *n* = 30	MDD	Sahaj yoga	30 minutes, 3×/week, 8-week total	TAU
Sharma et al. (2006), India	RCT, parallel; *n* = 30	MDD	Sahaj yoga	30 minutes, 3×/week, 8-week total	TAU
Sharma et al. (2016), USA	RCT, waitlist control; *n* = 25	MDD	SKY	90 minutes, 1×/week and 20–25-minute (home practice), daily, 8-week total	Waitlist control
Streeter et al. (2017), USA	Randomized controlled dosing study, parallel; *n* = 30	MDD	Iyengar yoga and coherent breathing (high-dose group)	90 minutes, 3×/week and 30-minute (home practice), 4×/week, 12-week total	Iyengar yoga + coherent breathing (low-dose group)
Streeter et al. (2020), USA	Randomized controlled dosing study, parallel; *n* = 28	MDD	Iyengar yoga and coherent breathing (high-dose group)	90 minutes, 3×/week + 30 minutes (home practice), 4×/week, 12-week total	Iyengar yoga + coherent breathing (low-dose group)
Talukdar et al. (2023), India	RCT, waitlist control; *n* = 33	MDD	Yoga therapy (combination)	52 minutes, 4–6×/week, 12-week total	Waitlist control
Tolahunase et al. (2018), India	RCT, parallel; *n* = 178	MDD	YMLI	120 minutes, 5×/week, 12-week total	SSRI treatment (drug therapy)
Tolahunase et al. (2018), India	RCT, parallel; *n* = 58	MDD	YMLI	120 minutes, 5×/week, 12-weeks total	TAU
Uebelacker et al. (2017), USA	RCT, parallel; *n* = 122	MDD	Hatha yoga	80 minutes, 1–2×/week, 10-week total	Psychoeducation
Weinstock et al. (2016), USA	RCT, parallel; *n* = 18	Bipolar disorder	Hatha yoga	80 minutes, 1–2×/week, 10-week total	Bibliotherapy
West et al. (2021), USA	RCT, parallel; *n* = 110	MDD	Hatha yoga	80 minutes, 1–2×/week, 10-week total	Psychoeducation

TAU: treatment as usual; MYI: mindful yoga intervention; CBT: cognitive behavioural therapy; MDD: major depressive disorder; SKY: Sudarshan Kriya yoga; YMLI: Yoga-based lifestyle intervention; RCT: randomized controlled trial.

**Table 2.** Summary of results.

Author (year)	Intervention	Outcome measure	Results	CASP grade
Bieber et al. (2021)	Ashtanga-yoga + TAU	1. BDI-II 2. MADRS	1. Significant mean difference found in BDI-II values for all participants, indicating Ashtanga yoga improves depressive symptoms. 2. The yoga group showed a significant symptom reduction of MADRS values over time compared to the control group.	7
Brinsley et al. (2022)	Yin yoga + Vinyasa yoga + aerobic exercise (stationary cycling) + stretching	1. POMS-TMD 2. POMS-depression	1. Reduction in POMS-TMD scores observed for all conditions in comparison to the control group. 2. POMS depression scores improved pre-post for all conditions except control.	8
Butler et al. (2008)	Hatha yoga + psychoeducation, group therapy + hypnosis + psychoeducation	1. CDRS 2. HRSD	1. Dysthymia symptom levels declined over time in each group with the exception of the first and second follow-ups for the control group. 2. Depressive symptom levels declined over time in each group.	5
Field et al. (2013)	Yoga (unspecified)	1. The CES-D 2. STAI	Both yoga and social support groups post-partum had decreased CES-D scores and anxiety on STAI.	6
Halappa et al. (2018)	Yoga therapy alone (unspecified), yoga therapy + medication	RAVLT	Significant improvement in performance on RAVLT was observed at follow-up in the yoga alone and yoga and medication groups in comparison to the control group.	1
Vollbehr et al. (2022)	MYI + mindful instructions	1. HRSD 2. DASS-sf	Both HRSD and DASS-sf difference scores showed improvement at 12-month follow-up for treatment as usual and MYI group.	7
La Rocque et al. (2021)	Bikram yoga	1. HAM-D 2. RRS	1. HAM-D scores significantly decreased in yoga and exercise groups but increased in the waitlist group. 2. RRS post-treatment scores were significantly lower in yoga and exercise groups compared to the waitlist group.	10
O’Shea et al. (2023)	CBT + therapeutic yoga	1. Depression Anxiety Stress Scales-21 (depression) 2. Depression Anxiety Stress Scales-21 (anxiety)	CBT and yoga group showed significantly lower levels of depression compared to CBT only group at 12-month follow-up. CBT and yoga groups maintained significant reductions in both anxiety and depression at 12-month follow-up in comparison to the CBT group.	5
Ravindran et al. (2021)	Breathing-focused yoga (unspecified)	1. MADRS 2. BDI-II	1. The yoga group experienced a significant decrease in MADRS score, while the psychoeducation group did not. 2. BDI-II significantly decreased from baseline in the yoga group.	11
Schuver and Lewis (2016)	Mindfulness-based yoga + mindfulness telephone sessions	BDI	Significant decrease in BDI scores and depressive symptoms observed in both treatment and control groups from baseline to post-intervention and 1-month follow-up.	8
Scott et al. (2019)	Iyengar yoga + coherent breathing (high-dose group)	1. PST 2. STAI-State	Both high-dose and low-dose groups experienced significant increases in positive emotions and decreased anxiety symptoms.	7
Sharma et al. (2006)	Sahaj toga	1. HAM-D 2. HAM-A	Both groups showed a decrease in HAM-D and HAM-A scores; however, participants engaging in Sahaj yoga experienced a greater reduction in scores.	7
Sharma et al. (2006)	Sahaj yoga	1. HAM-D 2. Letter cancellation test	The Sahaj yoga group showed greater improvements in HAM-D scores and executive functioning compared to the treatment-as-usual group.	5
Sharma et al. (2016)	SKY	HDRS-17	SKY intervention as an adjunct to pharmacotherapy demonstrated a greater mean reduction in HDRS scores.	8
Streeter et al. (2017)	Iyengar yoga and coherent breathing (high-dose group)	The BDI-II	Depression scores (BDI-II) decreased significantly in both the high-dose and low-dose groups.	9
Streeter et al. (2020)	Iyengar yoga and coherent breathing (high-dose group)	The BDI-II	Both the high-dose and low-dose groups experienced a significant reduction in BDI-II scores.	8
Talukdar et al. (2023)	Yoga therapy (combination)	Oxidative stress markers (MDA)	Participants in the yoga therapy group showed a significant decrease in plasma MDA levels.	5
Tolahunase et al. (2018)	YMLI	The BDI-II	Participants in the yoga group had a greater significant decrease in BDI-II scores compared to the control group.	7
Tolahunase et al. (2018)	YMLI	The BDI-II	The yoga group experienced a significant reduction in scores post-intervention compared to the control group.	8
Uebelacker et al. (2017)	Hatha yoga	QIDS	Both the yoga and psychoeducation groups showed a decrease in QIDS scores. However, the yoga group demonstrated lower depression severity at follow-up.	10
Weinstock et al. (2016)	Hatha yoga	QIDS-C	Depression symptoms decreased significantly for the yoga group. Both the yoga and bibliotherapy groups experienced similar levels of improvement in depression and quality of life scores.	9
West et al. (2021)	Hatha yoga	1. FFMQ 2. RSQ	1. The yoga group experienced positive changes in the ‘observe’ and ‘awareness’ facets of the FFMQ compared to the psychoeducation group. 2. There were no significant between- and within-group differences for rumination (RSQ scores).	10

TAU: treatment as usual; BDI-II: Beck Depression Inventory-II; MADRS: Montgomery–Asberg Depression Rating Scale; POMS: profile of mood states; TMD: total mood disturbance; CDRS: Cornell Dysthymia Rating Scale-Self Report; HRSD: Hamilton Rating Scale for Depression; CES-D: Center for Epidemiological Studies Depression Scale; STAI: State Anxiety Inventory; RAVLT: Rey auditory verbal learning test; MYI: mindful yoga intervention; DASS-sf: Depression scale of the Depression Anxiety Stress Scales-short form; HAM-D: Hamilton Rating Scale for Depression; RRS: Ruminative Response Scales; CBT: cognitive behavioural therapy; PST: positivity self-test; STAI-State: Spielberg State-Trait Anxiety Inventory State; HAM-A: Hamilton Rating Scale for Anxiety; SKY: Sudarshan Kriya yoga; HDRS-17: Hamilton Depression Rating Scale; MDA: malondialdehyde; YMLI: Yoga-based lifestyle intervention; QIDS: Quick Inventory of Depression Symptomatology; QIDS-C: Quick Inventory of Depression Symptomatology, Clinician Rating; FFMQ: Five Facet Mindfulness Questionnaire; RSQ: Response Styles Questionnaire; CASP: Critical Appraisal Skills Programme.

#### Symptom improvement

Table 2 presents the summary of the results of the included articles. From the 22 articles, 19 measured the effect of yoga on the severity of symptoms in participants with a mood disorder. All 19 studies demonstrated improvements in symptoms due to yoga; however, 3 of the studies (Field et al., 2013; Schuver and Lewis, 2016; Vollbehr et al., 2022) found the effect of yoga on the participants’ symptoms to be similar to that of the comparator groups (i.e. support groups, mindfulness and treatment as usual).

#### Dosing studies

Three of the 19 articles were also dosing studies that found both the low-dose and high-dose groups to improve mood disorder symptoms significantly (Scott et al., 2019; Streeter et al., 2017, 2020).

#### Cognitive outcomes

Two of the 22 studies evaluated yoga’s effect on cognition wherein the yoga groups demonstrated improved scores in outcome measures that evaluated auditory verbal learning and executive functioning (Halappa et al., 2018; Sharma et al., 2006).

#### Biomarker effects

Lastly, one study examined the effect of yoga on oxidative stress levels and reported a significant decrease in plasma malondialdehyde levels, thus suggesting an immunomodulatory effect of yoga on mood disorders (Talukdar et al., 2023).

### Risk of bias in studies

Supplemental Appendix B displays the CASP scores of the individual studies included, and Supplemental Appendix C shows the summary of the risk of bias for each question from the CASP checklist. Eleven of the selected studies had a low overall risk of bias, 10 studies had an unclear risk of bias, and 1 study had a high risk of bias. The average CASP score calculated was 7.32, which lies between the parameters of unclear and low risk of bias, thereby indicating some uncertainty regarding the quality and rigour of the included studies. The interpretation of CASP scores reflects as modified scoring system developed by the authors that is consistent with previous systematic reviews (Barr and Tsai, 2021; Bertini et al., 2022).

### Results of syntheses

**Figure 2.**
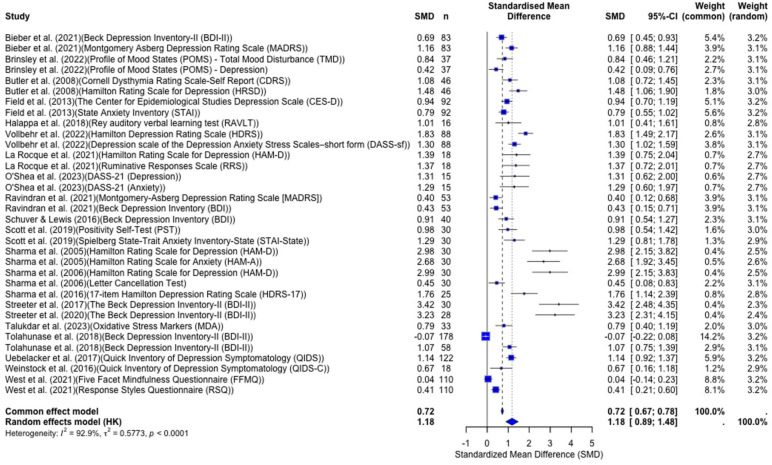
Forest plot.

From the 22 articles, 34 outcomes (primary and secondary) were identified and grouped together in one column, as shown in the forest plot in Figure 2. In relation to the comparator group, yoga interventions demonstrated a very large effect on the measured outcomes (SMD = 1.18, 95% CI [0.89, 1.48]). Two of the 34 measured outcomes did not have a statistically significant effect size (SMD = −0.07, 95% CI [−0.22, 0.08]; SMD = 0.04, 95% CI [−0.14, 0.23]; Tolahunase et al., 2018; West et al., 2021), potentially due to slight differences in intervention delivery or baseline participant characteristics. The remainder of the results indicated yoga to have a statistically significant positive effect on the measured outcomes. Heterogeneity was considerable among the included studies with a Higgins *I*^2^ value of 92.9% (95% CI [0.91, 0.94]) and a τ^2^ value of 0.58 (95% CI [0.38, 1.21]).

### Reporting biases

**Figure 3.**
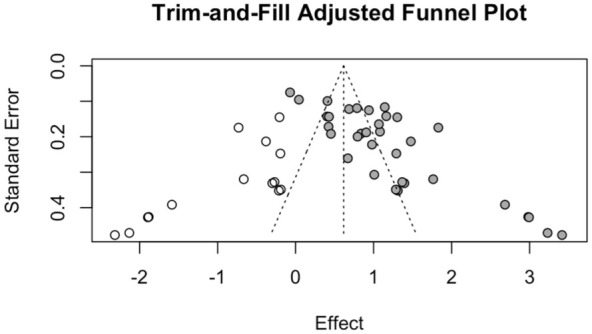
Trim-and-fill adjusted funnel plot.

A trim-and-fill analysis and fail-safe N calculations were conducted to complete a sensitivity analysis and account for publication bias. The trim-and-fill method imputed 14 potentially missing studies on the left side of the funnel plot corresponding to negative effects. Heterogeneity among the studies remained considerable (*I*^2^ = 94.2%, 95% CI [0.91, 0.94]; τ^2^ = 1.48, 95% CI [1.04, 2.56]). Furthermore, the exact fail-safe N value was calculated to be 1137. The exact fail-safe N analysis calculated that 1137 unpublished articles with a null effect would be required to reduce the overall effect size to a statistically non-significant level. Therefore, despite the potential publication bias observed by the trim-and-fill method, the high exact fail-safe N value suggests that the overall findings are unlikely to be nullified or overturned by unpublished articles. The trim-and-fill adjusted funnel plot is shown in Figure 3.

## Discussion

This systematic review aimed to evaluate the current literature on yoga and mood disorders, identifying the potential use of yoga in occupational therapy practice to manage symptoms and support occupational participation in adults experiencing mood disorders. The meta-analysis revealed considerable heterogeneity between the included studies. Due to this, the random effects model was used to generate a pooled SMD, which presented yoga as having a large effect size as an intervention in the included articles. These findings suggest that yoga has a moderate-to-large effect on the measured outcomes (mood disorder symptoms, cognition, etc.). However, due to the considerable heterogeneity and unclear risk of bias as observed by the CASP scores, caution must be exercised when generalizing these findings.

The sensitivity analysis, completed using the trim-and-fill method, indicated that considerable heterogeneity persisted even after imputation. Furthermore, this analysis adjusted the pooled SMD, suggesting that publication bias may have inflated the observed effects of yoga in the published articles. However, even with the adjustment, yoga’s effect size was still found to be statistically significant, although it was now considered moderate. Therefore, this indicates that the beneficial effect of yoga on adults with mood disorders persists, even after accounting for potential publication bias.

The findings of this review align with the results from previously published systematic reviews. Three meta-analyses have found that yoga has a moderate effect on improving symptoms and reducing the severity of mood disorders (Cramer et al., 2013; Rhoads et al., 2023; Wu et al., 2023). Yoga was generally found to have a positive effect on reducing depression when compared to passive and/or active controls (Bridges and Sharma, 2017; Cramer et al., 2013; Jean et al., 2022; Martínez-Calderon et al., 2023; Moosburner et al., 2024). Similarly, several previous studies have found yoga to be effective in reducing symptoms of anxiety among adults (Gallagher et al., 2020; Javnbakht et al., 2009; Zoogman et al., 2019). Consistent with the current findings, previous studies also support yoga as an effective intervention for symptom management among adults with bipolar disorder. For example, Lavey et al. (2005) reported notable reductions in anger, hostility and fatigue.

While the current review found only one review in support of the efficacy of yoga as a method for stress management, several previous studies have found significant stress level reductions with regular practice of yoga; underscoring potential supplementary benefits of yoga for symptom management in adults with mood disorders (Chong et al., 2011; Pascoe and Bauer, 2015; Shohani et al., 2018; Wang and Szabo, 2020). Consistent with the current review, existing literature indicates that yoga has positive effects on cognition, particularly among older adults (Brenes et al., 2019; Chobe et al., 2020; Hoy et al., 2021). For example, Chobe et al. (2020) found that memory, attention, and executive functioning improved among an elderly population following yoga as an intervention. An investigation of yoga for symptom management in individuals with mild cognitive impairment and dementia also found positive effects on cognitive functioning, sleep, mood and neural connectivity, highlighting a potential broader scope for yoga-based interventions and rehabilitation in adults (Brenes et al., 2019). These findings are particularly helpful in the occupational therapy context, as cognitive improvements in memory, attention and executive functioning via yoga therapy can significantly benefit occupational outcomes, including work engagement (Tan et al., 2022) and job satisfaction (Giles et al., 2013). Furthermore, most systematic reviews reported concerns regarding the methodology and the quality of evidence of their included studies (Cramer et al., 2013, 2017; Jean et al., 2022; Martínez-Calderon et al., 2023; Moosburner et al., 2024; Rhoads et al., 2023).

### Implications for occupational therapy

The findings of this systematic review and meta-analysis support the use of yoga interventions for symptom management among adults with mood disorders and support occupational participation in adults experiencing mood disorders. For example, Schmid et al. (2019) found that yoga interventions decreased depressive symptoms and improved occupational performance, including greater work engagement and satisfaction with performance, indicating a key relationship between depression and work outcomes. Furthermore, Lee et al. (2021) found that work–life balance was significantly associated with depression, further demonstrating that a reduction in depressive symptoms would likely improve work outcomes, including work–life balance. Similarly, Liu et al. (2024) found a significant negative association between anxiety and work engagement, indicating that a reduction in anxiety would increase work engagement. Overall, our results and the literature review demonstrate that reducing symptoms of mood disorders could significantly improve occupational outcomes and participation in adults, underscoring the benefit of integrating yoga therapy into occupational practices.

In addition, yoga interventions may play a critical role in improving daily functioning as a coping mechanism for mental or physical stress, supporting individuals to adapt to a new lifestyle while managing ongoing health challenges (Chong et al., 2011; Pascoe and Bauer, 2015; Shohani et al., 2018; Wang and Szabo, 2020; Willmott et al., 2025); these findings are also consistent with the model of human occupation (Grajo et al., 2018). Similarly, the health effects of yoga can extend to improvements in general functioning, with increased engagement in daily activities that were impacted by mood disorder symptoms. For example, several studies have shown that self-care activities (Egede et al., 2009; Holzapfel et al., 2009) and community engagement (Achterberg et al., 2003; Painter et al., 2012) might be impacted by depression and anxiety, underscoring how yoga interventions can improve functional outcomes in several domains. Ultimately, the findings from this systematic review and meta-analysis have significant implications in occupational therapy contexts, informing several ways that yoga can be used not only for symptom management but also for reintegration and increased participation in the workplace and the greater social context.

### Limitations

A limitation of this systematic review is that our inclusion criteria required articles to be published in or translated into English, thereby potentially excluding relevant articles published in other languages. Additionally, there were concerns regarding the methodology of the individual studies included in our review, particularly due to the unclear risk of bias reported by the CASP assessment. For example, common methodological concerns were the lack of blinded raters and the lack of standardization regarding the yoga intervention description and methodology of the primary articles, which pose a limitation when interpreting the results.

Moreover, several key intervention characteristics (e.g. instructor qualifications, delivery format) were not consistently reported across the included studies, limiting the conclusions that can be drawn regarding the efficacy of different yoga interventions and their influence mood disorders. Such inconsistencies likely contribute to the heterogeneity seen across the included studies and limit the ability to draw reliable conclusions regarding the efficacy of yoga-based interventions for improving mood disorders. The results of this study should be interpreted with caution in occupational therapy practice.

Furthermore, several of the yoga interventions examined in the primary articles may not be feasible for adults experiencing mood disorders due to the intensive time commitments and high frequency of sessions which may pose barriers to implementation in clinical settings. Future research might consider shorter protocols, group- or community-based interventions to improve feasibility across different populations. Lastly, the articles in our review lacked an occupational therapy perspective as they measured potential barriers to occupational participation (i.e. mood disorder symptoms) rather than evaluating occupational participation and engagement directly. This represents a large gap in the literature and underscores the importance for future research to focus on a more direct relationship between yoga interventions and occupational outcomes.

### Implications

To enhance the reliability and generalizability of the findings, future studies should include more standardized yoga protocols and adopt more rigorous methodological designs. Additionally, future studies investigating the use of yoga in adults with mood disorders should incorporate an occupational therapy lens by integrating yoga into occupational therapy sessions or utilizing outcome measures, such as the Canadian Occupational Performance Measure, to directly assess occupational participation and/or daily functioning. In regard to clinical practice, due to the positive effects of yoga on adults with mood disorders noted in our review and the lack of serious harm reported by the included studies, yoga can be considered as a potential therapeutic intervention for this population.

## Conclusion

In conclusion, although concerns were raised regarding the quality of evidence due to the unclear risk of bias reported from the average CASP score and the potential publication bias observed in the sensitive analysis, this review provides moderate supporting evidence for the use of yoga as an intervention for adults experiencing mood disorders. Yoga was found to be particularly effective in improving various symptoms of mood disorders, which are common barriers to occupational participation among this population. Taken altogether, the findings of this study and the greater literature underscore how the effects of yoga therapy for symptom reduction can significantly improve occupational outcomes in a work-context and in general daily functioning. Overall, future studies examining the use of yoga interventions for adults with mood disorders should employ more rigorous methodologies (e.g. larger samples, standardized protocols) and directly investigate the impact of yoga on occupational participation to advance knowledge of yoga within occupational therapy practice and inform evidence-based practice.

**Key findings**

- Yoga interventions significantly improved mood disorder symptoms, with a moderate to large effect size.
- Improvements were also seen in cognition and oxidative stress, despite varying study quality.
- Yoga reduces barriers to occupational participation (e.g. work engagement, job satisfaction and functioning in Activities of Daily Living (ADLs))

**What the study has added?**

This review supports yoga’s effectiveness in improving mood disorder symptoms, cognition and physiological indicators, serving as a supplementary, holistic therapy to facilitate stress management, engagement in ADLs and overall well-being.

## Supplemental Material

sj-docx-1-bjo-10.1177_03080226251412253 – Supplemental material for Yoga as a modality in occupational therapy practice for adults experiencing mood disorders: A systematic review and meta-analysis Supplemental material, sj-docx-1-bjo-10.1177_03080226251412253 for Yoga as a modality in occupational therapy practice for adults experiencing mood disorders: A systematic review and meta-analysis by Tenzin Dolker, Tenzin Dolma, Adrianna Spoerel, Marley Cameron, Briana Bortolin, Anna Colebatch, Candice Crooks, Helena Toolsiedas, Javier Mencia Ledo, Bao-Zhu Stephanie Long, Sana Siddiqui, Raihana Premji, Alicia McDougall and Behdin Nowrouzi-Kia in British Journal of Occupational Therapy
