# Supplementary material for: Yoga as a modality in occupational therapy practice for adults experiencing mood disorders: A systematic review and meta-analysis
Source: Br J Occup Ther. 2026 Jan 30;89(9):571–84. doi: 10.1177/03080226251412253 (PMC13451673; doi:10.1177/03080226251412253)
Supplement: Supplementary file 1 [file sj-docx-1-bjo-10.1177_03080226251412253.docx]

**Appendix A**

Search strategies

**OVID: Medline**

1. Mood Disorders/

2. Bipolar Disorder/

3. Cyclothymic Disorder/

4. depressive disorder, major/ or dysthymic disorder/ or premenstrual dysphoric disorder/

5. Yoga/

6. bipolar.tw,kf.

7. ((cyclothymi* or mood or affective) adj2 (disorder* or syndrome*)).tw,kf.

8. (major depress* adj2 (disorder* or syndrome*)).tw,kf.

9. (persistent depress* adj3 (disorder* or syndrome*)).tw,kf.

10. dysthymi*.tw,kf.

11. (premenstrual adj3 (syndrome or dysphori*)).tw,kf.

12. yoga.tw,kf.

13. yogic.tw,kf.

14. MDD.tw,kf.

15. 1 or 2 or 3 or 4 or 6 or 7 or 8 or 9 or 10 or 11 or 14

16. 5 or 12 or 13

17. 15 and 16

**OVID: Embase**

1. Mood Disorders/

2. Bipolar Disorder/

3. Cyclothymic Disorder/

4. depressive disorder, major/ or dysthymic disorder/ or premenstrual dysphoric disorder/

5. Yoga/

6. bipolar.tw,kf.

7. ((cyclothymi* or mood or affective) adj2 (disorder* or syndrome*)).tw,kf.

8. (major depress* adj2 (disorder* or syndrome*)).tw,kf.

9. (persistent depress* adj3 (disorder or syndrome)).tw,kf.

10. dysthymi*.tw,kf

11. (premenstrual adj3 (syndrome or dysphori*)).tw,kf.

12. MDD.tw,kf

13. yoga.tw,kf.

14. yogic.tw,kf.

15. 1 or 2 or 3 or 4 or 6 or 7 or 8 or 9 or 10 or 11 or 12

16. 5 or 13 or 14

17. 15 and 16

**OVID: APA PsycInfo**

1. Mood Disorders/

2. Bipolar Disorder/

3. Cyclothymic Disorder/

4. depressive disorder, major/ or dysthymic disorder/ or premenstrual dysphoric disorder/

5. Yoga/

6. bipolar.ti,ab.

7. (major depress* adj2 (disorder* or syndrome*)).ti,ab.

8. (persistent depress* adj3 (disorder or syndrome)).ti,ab.

9. ((cyclothymi* or mood or affective) adj2 (disorder* or syndrome*)).ti,ab.

10. dysthymi*.ti,ab.

11. (premenstrual adj3 (syndrome or dysphori*)).ti,ab.

12. MDD.ti,ab.

13. yoga.ti,ab.

14. yogic.ti,ab.

15. 1 or 2 or 3 or 4 or 6 or 7 or 8 or 9 or 10 or 11 or 12

16. 5 or 13 or 14

17. 15 and 16

**Cinahl Plus**

S1. (MH "Affective Disorders")

S2. (MH "Bipolar Disorder+")

S3. (MH "Cyclothymic Disorder")

S4. (MH "Dysthymic Disorder")

S5. (MH "Premenstrual Dysphoric Disorder")

S6. (MH Yoga)

S7. TI MDD AND AB MDD

S8. TI ( “mood disorder”* OR “affective disorder”* ) OR AB ( “mood disorder”* OR “affective

disorder”* )

S9. TI “bipolar disorder”* OR AB “bipolar disorder”*

S10. TI “Cyclothymic Disorder”* OR AB “Cyclothymic Disorder”*

S11. TI ( depress* N2 (major and disorder*) ) OR AB ( depress* N2 (major and disorder*)

S12. TI ( “persistent depress”* N2 (disorder* or syndrome) OR Dysthymi* N2 (disorder* or

syndrome) ) OR AB ( “persistent depress”* N2 (disorder* or syndrome) OR Dysthymi* N2

(disorder* or syndrome) )

S13. TI ( premenstrual dysphori* N3 (syndrome or disorder) ) OR AB ( premenstrual dysphori*

N3 (syndrome or disorder))

S14. TI ( yoga or yogic ) OR AB ( yoga or yogic )

S15. S1 OR S2 OR S3 OR S4 OR S5 OR S7 OR S8 OR S9 OR S10 OR S11 OR S12 OR S13

S16. S6 OR S14

S17. S15 AND S16

**Cochrane Library (Wiley)**

1. [mh "mood disorder"]

2. [mh "Bipolar Disorder"]

3. [mh ^"Cyclothymic Disorder"]

4. [mh ^"major depressive disorder"]

5. [mh ^"persistent depressive disorder"] OR [mh ^"dysthymic disorder"]

6. [mh ^"premenstrual dysphoric disorder"]

7. [mh ^yoga] OR [mh ^yogic]

8. (MDD):ti,ab,kw

9. ("mood disorder"*):ti,ab,kw

10. (bipolar disorder*):ti,ab,kw

11. (cyclothymic) NEAR/2 (disorder* or syndrome):ti,ab,kw

12. (depress* NEAR/2 ((major and disorder*) or syndrome)):ti,ab,kw

13. (persistent depress* NEAR/3 (disorder* or syndrome)):ti,ab,kw

14. (dysthymi*):ti,ab,kw

15. (premenstrual NEAR/3 (syndrome or dysphori*)):ti,ab,kw

16. (yoga):ti,ab,kw

17. (yogic):ti,ab,kw

18. #1 or #2 or #3 or #4 or #5 or #6 or #8 or #9 or #10 or #11 or #12 or #13 or #14 or #15

19. #7 or #16 or #17

20. #18 and #19

**Scopus**

1. TITLE-ABS-KEY ("mood disorders")

2. TITLE-ABS-KEY ("bipolar disorders")

3. TITLE-ABS-KEY ("cyclothymic disorder")

4. TITLE-ABS-KEY (("depressive disorder" AND major) OR ("dysthymic disorder") OR

("premenstrual dysphoric disorder"))

5. TITLE-ABS-KEY (yoga)

6. TITLE-ABS-KEY (bipolar)

7. TITLE-ABS-KEY (((cyclothymi* OR mood OR affective) W/2 (disorder* OR

syndrome*)))

8. TITLE-ABS-KEY **(**(major AND (depress* W/2 (disorder* OR syndrome* ))))

9. TITLE-ABS-KEY (( persistent AND (depress* W/2 (disorder* OR syndrome* ))))

10. TITLE-ABS-KEY (dysthymi*)

11. TITLE-ABS-KEY ((premenstrual W/3 (syndrome OR dysphori*)))

12. TITLE-ABS-KEY (yogic)

13. TITLE-ABS-KEY (mdd)

14. TITLE-ABS-KEY (yoga) OR (yogic)

15. (TITLE-ABS-KEY("mood disorders")) OR ("bipolar disorders")) OR

(TITLE-ABS-KEY("Cyclothymic Disorder")) OR (TITLE-ABS-KEY(("depressive disorder",

major) OR ("dysthymic disorder") OR ("premenstrual dysphoric disorder"))) OR

(TITLE-ABS-KEY(bipolar)) OR (TITLE-ABS-KEY(((cyclothymi* or mood or affective) W/2

(disorder* or syndrome*)))) OR (TITLE-ABS-KEY((major depress* W/2 (disorder* or

syndrome*)))) OR (TITLE-ABS-KEY((persistent depress* W/2 (disorder* or syndrome*)))) OR

(TITLE-ABS-KEY(dysthymi*)) OR (TITLE-ABS-KEY((premenstrual W/3 (syndrome or

dysphori*)))) OR (TITLE-ABS-KEY(MDD))

16. ((TITLE-ABS-KEY(Yoga)) OR (TITLE-ABS-KEY(yogic))) AND

((TITLE-ABS-KEY("mood disorders")) OR (TITLE-ABS-KEY("bipolar disorders")) OR

(TITLE-ABS-KEY("Cyclothymic Disorder")) OR (TITLE-ABS-KEY(("depressive disorder",

major) OR ("dysthymic disorder") OR ("premenstrual dysphoric disorder"))) OR

(TITLE-ABS-KEY(bipolar)) OR (TITLE-ABS-KEY(((cyclothymi* or mood or affective) W/2

(disorder* or syndrome*)))) OR (TITLE-ABS-KEY((major depress* W/2 (disorder* or

syndrome*)))) OR (TITLE-ABS-KEY((persistent depress* W/2 (disorder* or syndrome*)))) OR

(TITLE-ABS-KEY(dysthymi*)) OR (TITLE-ABS-KEY((premenstrual W/3 (syndrome or

dysphori*)))) OR (TITLE-ABS-KEY(MDD))

**Our search strat 15 & 16**

15. (TITLE-ABS-KEY("mood disorders")) OR (TITLE-ABS-KEY("bipolar disorders")) OR (TITLE-ABS-KEY("Cyclothymic Disorder")) OR (TITLE-ABS-KEY(("depressive disorder", AND major) OR ("dysthymic disorder") OR ("premenstrual dysphoric disorder")) OR (TITLE-ABS-KEY(bipolar)) OR (TITLE-ABS-KEY(((cyclothymi* or mood or affective) W/2 (disorder* or syndrome*)))) OR (TITLE-ABS-KEY((major AND (depress* W/2 (disorder* or syndrome*))))) OR (TITLE-ABS-KEY((persistent AND (depress* W/2 (disorder* or syndrome*))))) OR (TITLE-ABS-KEY(dysthymi*)) OR (TITLE-ABS-KEY((premenstrual W/3 (syndrome or dysphori*)))) OR (TITLE-ABS-KEY(MDD))

16. (TITLE-ABS-KEY(Yoga)) OR (TITLE-ABS-KEY(yogic)) AND

(TITLE-ABS-KEY("mood disorders")) OR (TITLE-ABS-KEY("bipolar disorders")) OR

(TITLE-ABS-KEY("Cyclothymic Disorder")) OR (TITLE-ABS-KEY(("depressive disorder",

major) OR ("dysthymic disorder") OR ("premenstrual dysphoric disorder"))) OR

(TITLE-ABS-KEY(bipolar)) OR (TITLE-ABS-KEY(((cyclothymi* or mood or affective) W/2

(disorder* or syndrome*)))) OR (TITLE-ABS-KEY((major AND (depress* W/2 (disorder* or

syndrome*))))) OR (TITLE-ABS-KEY((persistent AND (depress* W/2 (disorder* or syndrome*))))) OR (TITLE-ABS-KEY(dysthymi*)) OR (TITLE-ABS-KEY((premenstrual W/3 (syndrome or dysphori*)))) OR (TITLE-ABS-KEY(MDD))

**Appendix B**

Traffic-light Plot of the Included Studies

**Appendix C**

Summary Plot: Overall Risk of Bias
